# The Proteins PDIP3 and ZC11A Associate with the Human TREX Complex in an ATP-Dependent Manner and Function in mRNA Export

**DOI:** 10.1371/journal.pone.0043804

**Published:** 2012-08-23

**Authors:** Eric G. Folco, Chung-Sheng Lee, Kobina Dufu, Tomohiro Yamazaki, Robin Reed

**Affiliations:** Department of Cell Biology, Harvard Medical School, Boston, Massachusetts, United States of America; University of Cambridge, United Kingdom

## Abstract

The conserved TREX complex, which contains UAP56, Aly, CIP29, and the multi-subunit THO complex, functions in mRNA export. Recently, several putative new components of the human TREX complex were identified by mass spectrometry. Here, we investigated the function of two of these, PDIP3 and ZC11A. Our data indicate that both of these proteins are components of a common TREX complex and function in mRNA export. Recently, we found that both CIP29 and Aly associate with the DEAD box helicase UAP56 and with the TREX complex in an ATP-dependent manner. We now show that this is also the case for PDIP3 and ZC11A. Thus, together with previous work, our data indicate that the TREX complex participates in multiple ATP-dependent interactions.

## Introduction

During gene expression, pre-mRNAs are synthesized in the nucleus, undergo RNA processing, followed by export of the mature mRNA to the cytoplasm for translation. The TREX complex, which is conserved from yeast to human, functions in mRNA export [1], [2], [3]. The known components of the conserved TREX complex are UAP56, Aly, CIP29, and the multi-component THO complex [4], [5], [6]. Both CIP29 and Aly interact with the DEAD box helicase UAP56 in an ATP-dependent manner and require ATP for recruitment to TREX via UAP56 [4]. Recent mass spectrometry studies of immunopurified human TREX revealed five additional putative new components that appear to be unique to the metazoan TREX complex [4]. These are ZC11A, PDIP3, ELG, SRAG, and ERH.

Here we investigated the function of two of the putative new human TREX components, PDIP3 and ZC11A. We show that both proteins are immunoprecipitated (IP’d) by antibodies to known TREX components in RNase-treated nuclear extracts, and PDIP3 and ZC11A reciprocally co-IP in these extracts. Functional studies indicate that both PDIP3 and ZC11A function in mRNA export. Surprisingly, we found that both PDIP3 and ZC11A, like CIP29 and Aly, require ATP for association with UAP56 and the TREX complex. These data indicate that multiple ATP-dependent interactions are involved in TREX complex assembly.

**Figure 1:**
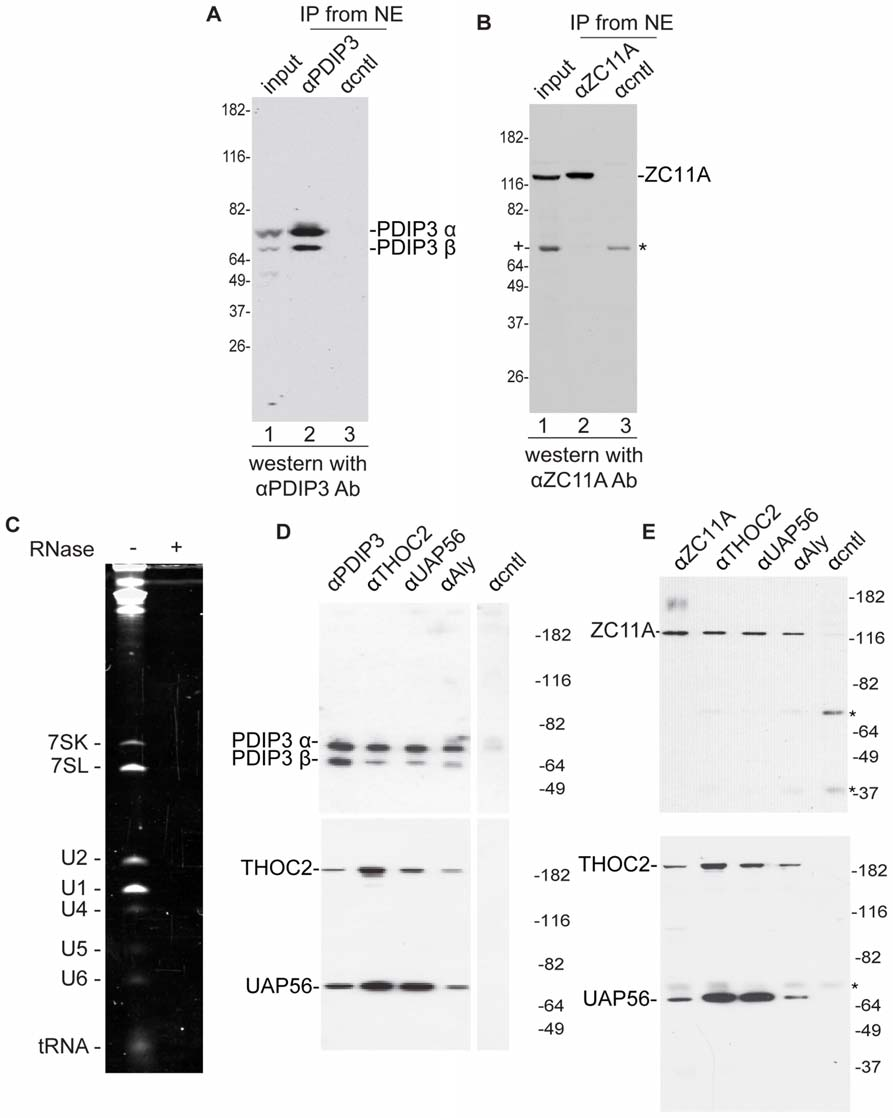
PDIP3 and ZC11A associate with the TREX complex. A, B. An antibody against PDIP3 (A) or ZC11A (B) recognizes their respective antigens on a Western blot, and IPs them from nuclear extract. We note that in the input lane ZC11A antibodies detect another band (designated by +), which may be non-specific. 10% of the input was loaded for the IPs. C, D, E. Nuclear extract was RNase-treated (C) and IPs were carried out with the indicated antibodies (D, E) incubated in the presence of ATP. Antibodies against the indicated proteins were used for Westerns. The bands designated by the asterisks are heavy and light chains of the antibody.

## Results and Discussion

### PDIP3 and ZC11A co-IP with TREX Components

To further characterize the human TREX complex, we sought to analyze the putative new components. PDIP3 was of particular interest because of its high similarity to Aly (∼40% identical) [7]. To characterize PDIP3, we raised a rabbit polyclonal antibody against a C-terminal peptide. This antibody recognizes the two major forms of PDIP, PDIPα and β, and IPs both forms (Fig. 1A). The α and β forms of PDIP3 are splice variants that are 46 and 43 kD, respectively, and both forms contain an RRM that is 42% identical to that of Aly [7]. To characterize ZC11A, we used a commercially available polyclonal antibody, which recognizes a protein of the correct size by Western, and this protein is specifically IP’d by the ZC11A antibody (Fig. 1B). ZC11A contains three amino terminal zinc fingers of the CCCH type and nothing else is known about this protein to our knowledge. To further investigate PDIP3 and ZC11A, we RNase-treated HeLa nuclear extracts (Fig. 1C) and used them for IP/Westerns (Fig. 1D, E). This analysis revealed that PDIP3α and β efficiently co-IP with TREX components, including THOC2, UAP56 and Aly (Fig. 1D). Previous work showed that PDIP3 (also known as Skar [7]) interacts with and is a substrate of S6K1 [7]. PDIP3 was also reported to associate with the exon junction complex (EJC), which is recruited to exon junctions during splicing [8], [9]. We did not identify a significant association between PDIP3 and the exon junction complex [4]. However, like its relative Aly, we found that PDIP3 is abundantly associated with TREX complex components (Fig. 1D). These differences in associations with the EJC and TREX complex may be due to the assay systems used and/or may indicate that a handoff of PDIP3 occurs between the TREX complex and the EJC during the mRNA export pathway. As observed with PDIP3, we also found that ZC11A efficiently co-IPs with TREX components in RNase-treated nuclear extracts (Fig. 1E).

**Figure 2:**
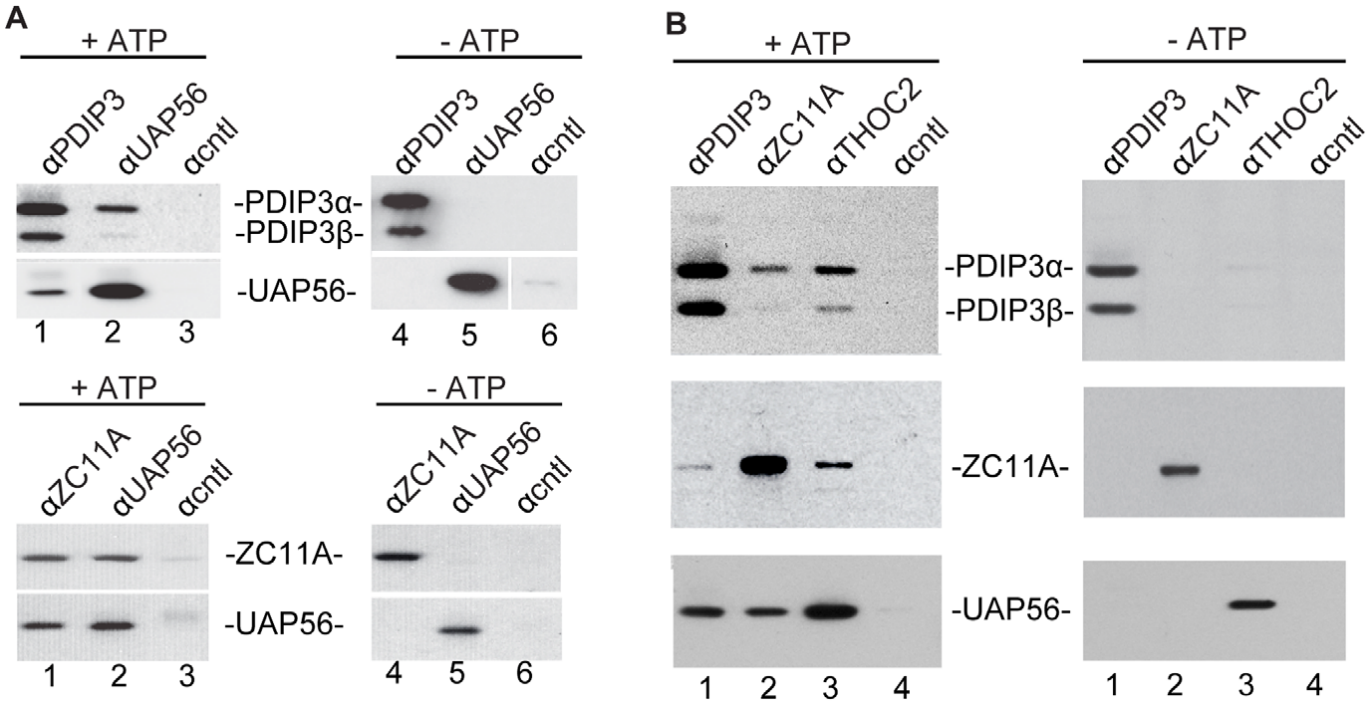
PDIP3 and ZC11A associate with UAP56 and the TREX complex in an ATP-dependent manner. A, B. IPs were carried out with the indicated antibodies using RNase-treated nuclear extract incubated in the presence or absence of ATP. Antibodies against the indicated proteins were used for Westerns.

### PDIP3 and ZC11A Associate with the TREX Complex in an ATP-dependent Manner

In recent work, we found that both Aly and CIP29 associate with UAP56 and the THO complex in an ATP-dependent manner [4]. In contrast, the THO complex associates with UAP56 in an ATP-independent manner [4]. For the IP/Westerns carried out in Figs. 1D and E, we included ATP in our nuclear extracts. Thus, we next sought to determine whether ATP affected the association of PDIP3 or ZC11A with UAP56. To do this, we incubated RNase-treated nuclear extract in the presence or absence of ATP, followed by IP/Westerns. Remarkably, this analysis revealed that both PDIP3 (Fig. 2A, top panels) and ZC11A (2A, bottom panels) associate with UAP56 in the presence, but not in the absence, of ATP. We next examined whether ATP affected the association between PDIP3, ZC11A, and the THO complex. Significantly, PDIP3, ZC11A, and THOC2 co-IP’d only in the presence of ATP (Fig. 2B). As expected from our previous work, the association between UAP56 and THOC2 was ATP-independent (Fig. 2B). Thus, together, our data indicate that both PDIP3 and ZC11A, like Aly and CIP29, interact with UAP56 and the THO complex in an ATP-dependent manner. Moreover, the observation that ZC11A and PDIP3 co-IP both with each other and with other TREX components suggests that these proteins form one common TREX complex.

**Figure 3:**
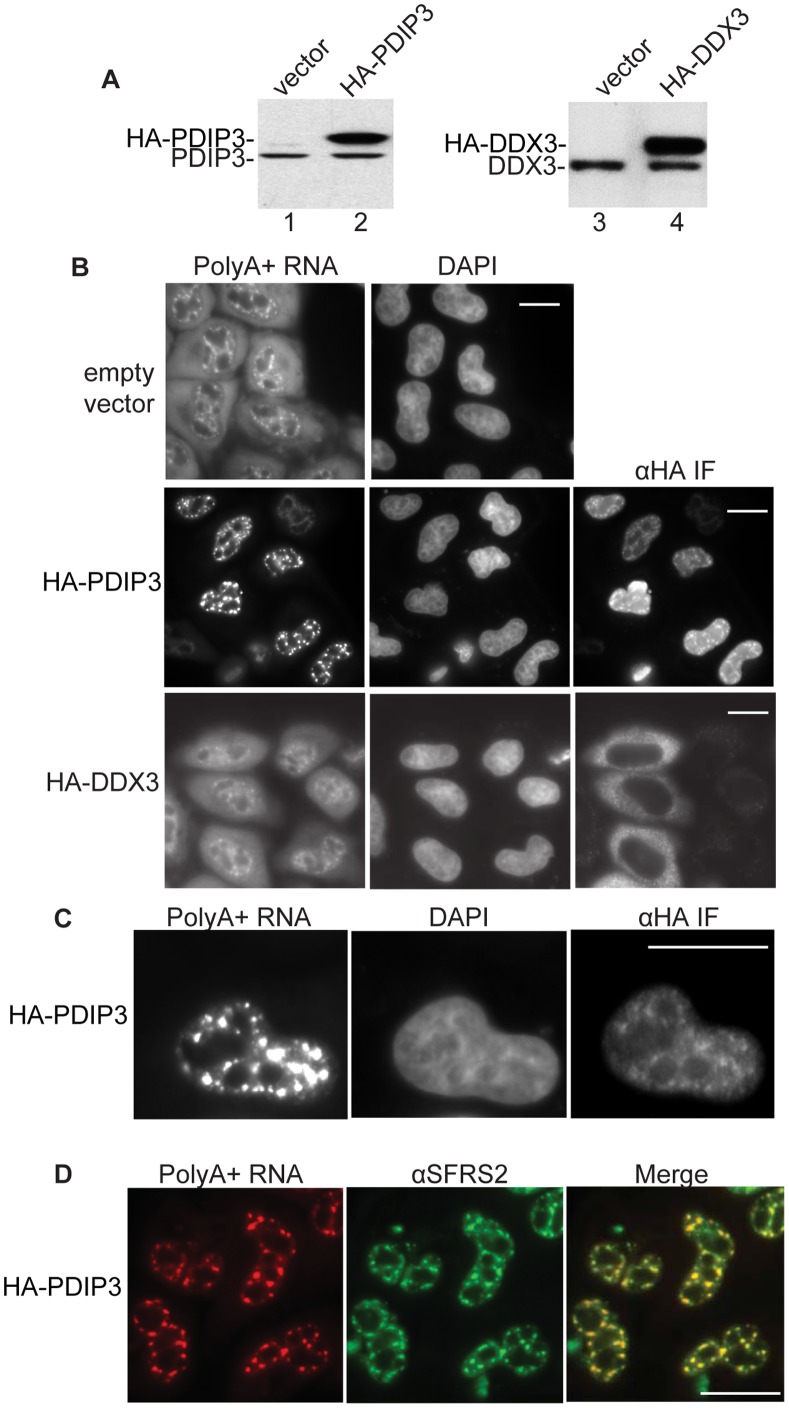
Overexpression of PDIP3 causes retention of polyA+ RNA in nuclear speckle domains. A. Westerns of HeLa cell lysates containing empty vector (lanes 1 and 3), overexpressing HA-PDIP3 (lane 2) or expressing HA-DDX3 (lane 4). B. Nucleocytoplasmic distribution of total polyA+ RNA in HeLa cells expressing empty vector, HA-PDIP3, or HA-DDX3. PolyA+ RNA was detected by FISH using an Alexa Fluor labeled oligo dT(70) probe. DAPI staining was used to visualize the nucleus and IF to detect the HA-tagged proteins. Scale bar, 10 µm. C. Higher magnification of a cell transfected with HA-PDIP3. Scale bar, 10 µm. D. PolyA+ RNA is retained in nuclear speckles in HeLa cells overexpressing HA-PDIP3. PolyA+ RNA was detected by FISH and nuclear speckles were detected by IF using an antibody to SFRS2. The merged image is shown. Scale bar, 10 µm.

### PDIP3 and ZC11A Function in mRNA Export

We next asked whether PDIP3 and ZC11A have roles in mRNA export. Although PDIP3 was efficiently knocked down using RNAi, we did not observe an export phenotype. We also knocked down Aly alone or in combination with PDIP3, as these two proteins are related. Consistent with previous work [10], [11], [12], a significant inhibition of polyA+ export was observed with the Aly knockdown alone, but this inhibition was not further exacerbated by the PDIP3 knockdown. Thus, to investigate whether PDIP3 plays a role in mRNA export, we overexpressed full length HA-tagged PDIP3 or a HA-tagged negative control protein (HA-DDX3). Western blots confirmed that the HA-tagged proteins were overexpressed (Fig. 3A). In addition, IF with HA antibodies showed that HA-PDIP3 is present in nuclear speckles domains (Fig. 3B), as observed with other known TREX components [2]. We then transfected HeLa cells with empty vector, HA-tagged PDIP3 or HA-tagged DDX3 and carried out fluorescent in situ hybridization (FISH) for total polyA+ RNA (Fig. 3B). DAPI was used to identify the nucleus and immunofluorescence (IF) was used to identify cells containing the overexpressed HA-tagged proteins (Fig. 3B). Significantly, this analysis revealed that polyA+ RNA export was potently inhibited in the cells containing overexpressed PDIP3, but was unaffected in cells containing the empty vector or the overexpressed negative control DDX3 (Fig. 3B). Moreover, higher magnification images of cells transfected with HA-PDIP3 revealed that the polyA+ RNA accumulated in discrete nuclear foci (Fig. 3C). In recent work, we found that polyA+ RNA accumulates in nuclear speckle domains when either UAP56 or Aly are knocked down [12]. As shown in Fig. 3D, polyA+ RNA also accumulates in nuclear speckle domains in PDIP3-overexpression cells, as the foci containing the polyA+ RNA co-localize with the nuclear speckle domain marker protein SFRS2. The observation that PDIP3 overexpression inhibits mRNA export suggests that this protein may be functioning as a dominant negative and thus may play a role in mRNA export. In addition, it is possible that, similar to other TREX components [12], PDIP3 functions in releasing polyA+ RNA from nuclear speckle domains. Alternatively, PDIP3 may retain polyA+ RNA in speckles because the mRNA accumulates together with PDIP3 in speckles when PDIP3 is overexpressed.

**Figure 4:**
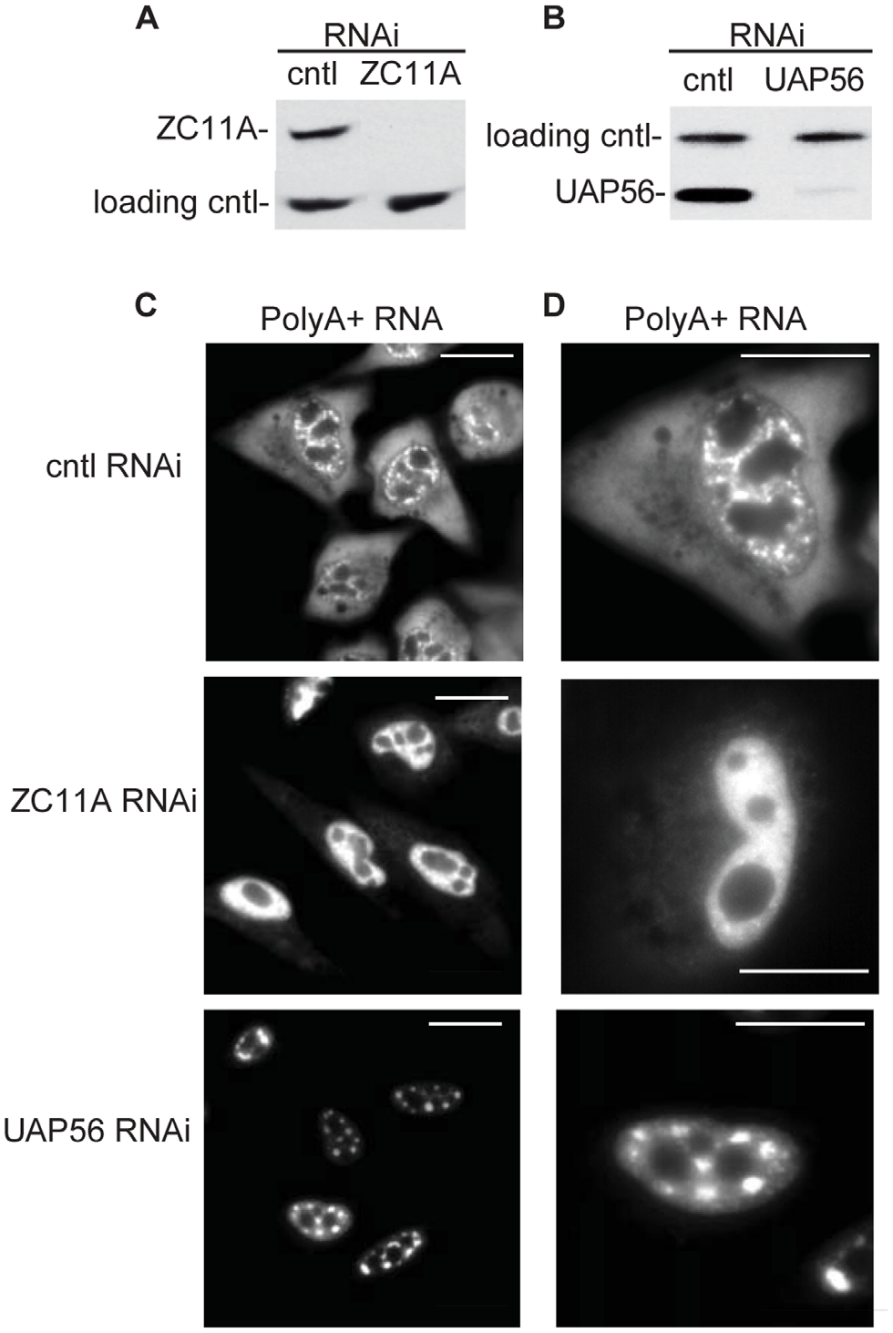
RNAi of ZC11A causes retention of polyA+ RNA in the nucleoplasm. A, B. The knockdown efficiency of ZC11A (A) or UAP56 (B) in HeLa cells was analyzed by Western. Loading controls were eIF4A3 (A) and CBP80 (B). Non-targeting siRNA was used as a control (cntl). C. Knockdown of ZC11A or UAP56/DDX39A results in retention of polyA+ RNA in the nucleus. PolyA+ RNA was visualized by FISH using an Alexa Fluor labeled oligo dT(70) probe. Scale bar, 10 µm. D. Same as C, except showing higher magnification of FISH images. Scale bar, 10 µm.

To determine whether ZC11A functions in mRNA export, we transfected HeLa cells with siRNAs targeting ZC11A. Non-targeting siRNA was used as a negative control, and siRNAs targeting UAP56 (and its close relative DDX39A) were used as a positive control. Western analysis showed that ZC11A and UAP56 were efficiently knocked down by their respective siRNAs but not by the negative control siRNA (Fig. 4A and B). We then carried out FISH for total polyA+ RNA in the knockdown cells. Significantly, this analysis revealed that mRNA export was abolished in the ZC11A knockdown cells, as observed with the UAP56 knockdown (Fig. 4C). In contrast, there was no effect on mRNA export with the negative control knockdown (Fig. 4C). To further characterize the ZC11A phenotype, we examined the cells under higher magnification (Fig. 4D). Unexpectedly, this analysis revealed that polyA+ RNA was evenly distributed throughout the nucleoplasm (note that the dark regions are nucleoli) rather than retained in nuclear speckles, as observed with knockdown of other TREX components (e.g. UAP56 and Aly). These data suggest that ZC11A functions at a different step in the mRNA export pathway than the factors that result in nuclear speckle retention.

In this study, we have characterized PDIP3 and ZC11A as two new components of the human TREX complex, and we have provided evidence that both proteins function in mRNA export. Remarkably, as we observed with Aly and CIP29 [4], both PDIP3 and ZC11A associate with UAP56 and the TREX complex in an ATP-dependent manner. In future studies it will be essential to understand the role of these numerous ATP-dependent interactions in TREX function. The observation that all of these proteins associate with a common TREX complex suggests that they remain in the TREX complex but may function in its dynamic remodeling during the mRNA export pathway.

## Materials and Methods

### Plasmids

The human PDIP3 gene was cloned into *XbaI* and *EcoRV* sites of pcDNA3.1 (Invitrogen). The HA tag nucleotide sequence was inserted at the 3′ end of the PDIP3 gene by PCR. The ZC11A gene was cloned into the Xba1 and EcoRV sites of pcDNA3.1 (−) Hygromycin (Invitrogen).

### Antibodies

The PDIP3 rabbit polyclonal antibody was raised against the C-terminal peptide sequence (NPPAEVDPDTILKALFKSSGAS (Genemed Synthesis)) from human PDIP3. The mouse polyclonal antibody against ZC11A and the mouse monoclonal antibody against SFRS2 were from Abnova and Sigma, respectively. Antibodies against TREX components and eIF4A3 were described [13], [14], [15], [16], [17]. The non-related M2 and SAP130 rabbit polyclonal antibodies were used as negative controls for Figures 1 and 2, respectively.

### IP/Westerns

Antibodies were coupled to protein A Sepharose and covalently cross-linked using dimethylpimelimidate (Sigma). Reaction mixtures (500 µl) containing or lacking ATP were prepared and used for the IPs. The +ATP reaction mixture contained 150 µl of HeLa nuclear extract, 150 µl of SDB (20 mM HEPES at pH 7.9, 100 mM KCl), 500 µM ATP, 3.2 mM MgCl_2_, 20 mM creatine phosphate, and 50 ng/µl RNase A and was incubated for 20 min at 30°C. The reaction mixture was then added to 500 µl of IP buffer (1X PBS, 0.1% Triton, 0.2 mM PMSF, protease inhibitor EDTA-free [Roche]) and 20 µl of antibody-cross-linked beads. IPs were carried out overnight at 4°C, followed by four 1.5 ml washes with IP buffer. Proteins were eluted with 25 µl of SDS loading buffer (0.65% Tris, 25% glycerol, 4.5% SDS, 0.005% Bromophenol Blue) followed by incubation for 20 min at room temperature. DTT was then added to a final concentration of 5 mM, the samples were boiled for 4 min, and 7 µl of each IP was fractionated on a 4%–12% SDS gradient gel. For IPs under -ATP conditions, ATP, MgCl_2_, and creatine phosphate were omitted and substituted with water.

### Cell Culture and Transfection

HeLa cells were cultured in DMEM supplemented with 10% FBS and 1% penicillin/streptomycin. HeLa cells were transfected on 35 mm dishes with glass coverslip bottoms (MatTek Corp., MA) using lipofectamine 2000 (Invitrogen). To overexpress HA-tagged proteins, 2 µg of plasmid DNA was transfected into cells that were 70% confluent for 24 hrs. For RNAi, HeLa cells were plated overnight until they were 40% confluent followed by transfection using lipofectamine 2000. For ZC11A, the siRNA mixture was from Dharmacon (catalog # siGENOME SMARTpool siRNA D-021238). The target sequences are 5′-GUGAAAGGGUGCAAGCGAA-3′, 5′-AGCUAAAGAUUGAUAGUGA-3′, 5′-GGUGACAUGAUUAGAGGAA-3′ and 5′-UGACAGUGAUCCUCCAUUA-3′ at an equal concentration. The siRNA target sequences for UAP56/DDX39A were described [18]. The control siRNA was purchased as non-targeting siRNA from Dharmacon (catalog # D-001810–04).

### FISH and IF

To detect polyA+ RNA by FISH, an HPLC-purified oligo dT(70) probe labeled at the 5′ end with Alexa Fluor 546 NHS Ester was used. HeLa cells were washed once with PBS, fixed for 10 min with 4% paraformaldehyde, washed three times with PBS, permeabilized with 0.5% Triton X-100 for 5 min, washed twice with PBS and then once with 2X SSC for 10 min at RT. The oligo dT(70) probe was added at 1 ng/µl followed by incubation for 16–24 hrs at 42°C. The cells were then washed at room temperature for 15 min each wash, twice with 2X SSC, once with 0.5X SSC and once with PBS. Images were captured with an EM-CCD camera on an inverted microscope (200M; Zeiss, Thornwood, NY) using Metamorph software (Molecular Devices, Sunnyvale, CA). Primary antibodies were against the HA tag (1∶200) and SFRS2 (1∶1000). The secondary antibody was mouse Alexa-488 (Invitrogen) diluted to 1∶1000 with IF solution (PBS, 0.1% Triton X100, 2 mg/ml RNase free BSA; Ambion).
